# Species specific anaesthetics for fish anaesthesia and euthanasia

**DOI:** 10.1038/s41598-017-06917-2

**Published:** 2017-08-02

**Authors:** Gareth D. Readman, Stewart F. Owen, Toby G. Knowles, Joanna C. Murrell

**Affiliations:** 1 School of Biological and Marine sciences, University of Plymouth, Plymouth, PL4 8AA United Kingdom; 2 AstraZeneca, Alderley Park, Macclesfield, Cheshire SK10 4TF United Kingdom; 3 School of Veterinary Science, University of Bristol, Langford House, Langford, Bristol BS40 5DU United Kingdom

## Abstract

There is a need to ensure that the care and welfare for fish maintained in the laboratory are to the highest standards. This extends to the use of anaesthetics for both scientific study, humane killing and euthanasia at end of life. An anaesthetic should not induce negative behaviours and fish should not seek to avoid the anaesthetic. Surprisingly little information is available to facilitate a humane choice of anaesthetic agent for fish despite over 100 years of use and the millions of fish currently held in thousands of laboratories worldwide. Using a chemotaxic choice chamber we found different species specific behavioural responses among four closely related fish species commonly held in the laboratory, exposed to three widely used anaesthetic agents. As previously found for zebrafish (*Danio rerio*), the use of MS-222 and benzocaine also appears to induce avoidance behaviours in medaka (*Oryzias latipes*); but etomidate could provide an alternative choice. Carp (*Cyprinus carpio*), although closely related to zebrafish showed avoidance behaviours to etomidate, but not benzocaine or MS-222; and rainbow trout (*Oncorhynchus mykiss*) showed no avoidance to the three agents tested. We were unable to ascertain avoidance responses in fathead minnows (*Pimephales promelas*) and suggest different test paradigms are required for that species.

## Introduction

Summaries in the journal *Nature* ^1, 2^ and also the report from the second Newcastle meeting on laboratory animal euthanasia^3^ have highlighted that the humane use of anaesthesia for fish is far more complicated than previously thought. The publication of two studies using different behavioural test paradigms: a chemotaxic chamber^4^ and conditioned place avoidance^5^ have demonstrated the avoidance of the Zebrafish (*Danio rerio*) to the commonly used anaesthetic Tricaine methanosulphate (MS 222). The European directive (2010/63 EU) article 14^6^, which covers the protection of animals used for scientific purposes, specifically states that any severe procedure should not be conducted without anaesthetic and that for other procedures the use of anaesthesia should be based on a utilitarian benefit commensurable with the aims of the procedure. For mammals, standard practice uses species specific choice of anaesthetic agent. This is to facilitate effective anaesthesia and to reduce negative behavioural responses to the anaesthetic agent in the early stages of induction. As different responses to anaesthetics are apparent even among relatively closely related mammalian species (e.g. mice and rats)^7^, it seems logical to ask if there are similar considerations for fish, *viz*. are there species specific requirements for anaesthetic agents in fish?

Assessment of the practical aspects of using different anaesthetics, for different fish species have been reviewed in several papers^8–13^. These reviews generally focus on ease of delivery, rapid onset and stability, and not the potential welfare implications associated with induction. To date, it appears that the welfare implication of each potential anaesthetic has not been considered on a species specific basis for fish. In fact it seems a ‘one size fits all’ approach is advocated by many^9^. It is clear that regardless of the time it takes to anaesthetise a fish, the first fundamental question should be *‘does it produce avoidance behaviour?’* Beyond that question the next should be ‘*does it act as an anaesthetic?*’ It is difficult to answer the second question conclusively without physiological and bioelectric evidence and we do not attempt to do so in this paper. The first question however is relatively simple. Indeed there are several approaches with which to answer this question^4, 5^; and the answer for one species (zebrafish) already appears to be that many commonly used anaesthetics provoke negative behavioural responses^4^, and most importantly that zebrafish appear to retain some memory of the event^5^. Therefore, there is clear evidence that humane choices for that species can now be made based on behavioural responses indicative of avoidance behaviour^4^.

There are likely hundreds of diverse species of fish used in scientific experiments globally. However, the range of species used in large numbers is somewhat more restricted. For example, commonly recommended species in Organisation for Economic Cooperation and Development (OECD) and Environmental Protection Agency (EPA) guidelines for use in regulatory testing represent some of the most commonly used species after zebrafish; and several of these species crossover into aquaculture research (carp (*Cyprinus carpio*), rainbow trout (*Oncorhynchus mykiss*), salmon (*Salmo salar*)). Here we chose four commonly used species; carp, fathead minnows (*Pimephales promelas*), medaka (*Oryzias latipes*) and rainbow trout. These species represent a range of those used in significant numbers in research, and their responses to the two most commonly used anaesthetics MS 222 and benzocaine were assessed. We also examined their response to etomidate which was previously shown not to provoke avoidance behaviours in Zebrafish^4^.

## Results

The parameter estimates produced by MLwiN (Table 1

**Table 1:** Summary of MLwiN estimates (±SEM) of the effects for each of the anaesthetic agents in dilution water versus exposure for the response variable Time.

Control (No compound)	Time in clean lane (secs)	Treatment lane difference (secs)
Carp	76.155 (7.421)	−9.890 (8.569)
Fathead Minnow	80.343 (14.308)	21.625 (16.522)
Medaka	96.190 (12.007)	−2.760 (13.865)
Rainbow trout	67.345 (9.065)	−8.820 (10.467)
**HCl**	**Time in clean lane (secs)**	**Treatment lane difference (secs)**
Carp	131.938 (7.952)	−97.695 (9.182)***
Fathead Minnow	83.168 (12.354)	−5.155 (14.265)
Medaka	101.130 (13.044	−61.290 (15.062)***
Rainbow trout	118.557 (6.088)	−64.555 (7.030)***
**Ethanol**	**Time in clean lane (secs)**	**Treatment lane difference (secs)**
Carp	81.942 (19.038)	−11.605 (11.481)
Fathead Minnow	74.652 (17.046)	−10.515 (19.683)
Medaka	79.223 (5.473)	−10.635 (6.319)
Rainbow trout	77.972 (7.689)	8.315 (8.878)
**Benzocaine**	**Time in clean lane (secs)**	**Treatment lane difference (secs)**
Carp	79.005 (13.831	14.530 (15.971)
Fathead Minnow	63.880 (17.480)	10.200 (20.184)
Medaka	101.638 (13.772)	−24.235 (8.305)**
Rainbow trout	99.680 (11.825)	−16.730 (13.654)
**Etomidate**	**Time in clean lane (secs)**	**Treatment lane difference (secs)**
Carp	82.618 (7.236)	−21.595 (8.355)**
Fathead Minnow	66.133 (16.463)	33.185 (19.010)
Medaka	68.933 (5.970)	4.625 (6.894)
Rainbow trout	54.585 (12.454)	18.140 (14.381)
**MS222**	**Time in clean lane (secs)**	**Treatment lane difference (secs)**
Carp	68.850 (11.199)	9.280 (12.932)
Fathead Minnow	52.700 (13.027)	47.460 (15.042)***
Medaka	88.027 (5.710)	−38.645 (6.594)***
Rainbow trout	95.652 (9.377)	−10.515 (10.828)

Statistical significance is denoted by *P < 0.05, **P < 0.01, ***P < 0.001.

) show the average time spent in the control lane compared with the time spent in the exposure lane and the statistical significance of any difference. For example, if we look at the analysis for Ethanol and choose carp as our species then the model shows that the average time spent in the exposure lane was 11.6 seconds less than that of the control value of 81.9 seconds, and that there was no significant difference in the time spent in either lane.

### Control exposures

No preference for the amount of time spent in either lane (right versus left) was demonstrated in the absence of test compounds for any of the species tested, demonstrating no environmental bias in the system.

### Positive control

Hydrochloric acid (HCl resulting in approximately pH 3) was used as a positive control to assess the functionality of the system and to provide a model of altered behavioural response. Of the species tested carp, medaka and rainbow trout all showed significant aversion to HCl (P < 0.001), since they spent significantly less time in the exposure versus treated lane. Fathead minnows showed no significant difference in time spent in either exposure or control lane (Table 1).

### Solvent control

None of the species tested showed difference in the time spent in exposure versus control lanes (Table 1), suggesting that ethanol was not aversive at the level used in this study (approx. 0.033% of final exposure). This lack of preference indicates that the use of ethanol at this concentration as a carrier solvent does not create an aversive response in itself.

### Benzocaine

Of the species tested only medaka showed a significant avoidance (P < 0.01) based on the time spent in exposure versus control lanes. Fathead minnow, carp and rainbow trout showed no significant difference in time spent in either lane (Table 1).

### Etomidate

Carp were the only species to show significant avoidance (P < 0.01) spending less time spent in exposure versus control lanes, fathead minnows, medaka and rainbow trout showed no significant difference between either lane (Table 1).

### MS 222

Medaka showed a significant difference in the time spent in exposure versus control lanes (P < 0.001) showing avoidance of MS 222. Carp and rainbow trout showed no significant difference in time spent in either lane (Table 1), while fathead minnows showed a significant preference for the lane containing the MS222 versus the control lane (P < 0.01).

### pH measurements

At no point during the exposure of any anaesthetic compounds did the pH values in the experimental or control lane differ by more than one pH unit, across each experimental group (n = 20).

Although routine practice is to buffer MS222 due to the reduction in pH caused by its addition, within this study the pH was not buffered due to the inherent capacity of the holding water (potable mains water treated to achieve OECD standards) resultant mean/min/max values for pH during exposure of MS222 to the four different species was as follows: Carp = 6.90/6.58/7.40, fathead minnow = 7.14/6.88/7.39, medaka = 7.10/6.93/7.35 and rainbow trout = 7.10/6.96/7.30.

## Discussion

A chemotoxic preference test with quantitative analysis (time) was used to assess the difference in a specific behaviour of four fish species to three commonly used anaesthetics. This method was used previously^4^ to examine the behavioural differences of a single species, zebrafish (*Danio rerio*), and define to the response to nine anaesthetics previously used for fish. There has been a little confusion and the work accidentally miss-cited^6^; for clarity the exposure concentrations tested in Readman *et al*.^4^ was to half the dose required for anaesthesia (Table 2), and metomidate [CAS 5377-20-8] was not tested for aversion and is different to etomidate [CAS 33125-97-2]^4^. However both substances are GABA modulating agents and a different mode of action to the sodium-channel blockers such as MS222. However, as with all pharmaceutical agents, the uptake, pharmacokinetics, receptor binding and activities will be different for each compound, and likely each species and will need to be compared head-to-head to assess any potential differences. The recommendation from our previous work for adult zebrafish was to suggest etomidate was the best option for routine anaesthesia^4^. As with all anaesthesia protocols attention should be paid not only to the appropriate species-specific compound used to achieve anaesthesia, but also to the aim of the protocol. Careful choice of the compound might allow synergistic or potential analgesic effects of the anaesthetic used post-operatively. Best practice could include a cost benefit analysis of the potential suffering caused at any point before, during or after the procedure.

**Table 2:** Test substance effective dosage and supplier details.

Species	Test substance	Effective published dose*	Test concentration	Reference	Supplier	Stock base
All	Baseline control (no exposure)	N/A	N/A	N/A	N/A	N/A
	Hydrochloric acid (+ve control) pH 3.0	N/A	N/A	N/A	Sigma - Aldrich	N/A
	Ethanol 99.8% (solvent control)	1 ml/L	1 ml/L	N/A	Sigma - Aldrich	N/A
Carp	Benzocaine	100 mg/L	50 mg/L	9, 10, 12, 22, 24	Sigma - Aldrich	Ethanol
	Etomidate	1 mg/L	0.5 mg/L	9, 10, 12, 25	Ark Pharm Inc	Ethanol
	MS222	100 mg/L	50 mg/L	9, 10, 12, 26, 27	Sigma - Aldrich	Water
Fathead Minnow	Benzocaine	100 mg/L	50 mg/L	9, 10, 12, 22, 24	Sigma - Aldrich	Ethanol
	Etomidate	4 mg/L	2 mg/L	10, 12, 28	Ark Pharm Inc	Ethanol
	MS222	75 mg/L	37.5 mg/L	10, 12, 28	Sigma - Aldrich	Water
Medaka	Benzocaine	100 mg/L	50 mg/L	9, 10, 12, 22	Sigma - Aldrich	Ethanol
	Etomidate	2 mg/L	1 mg/L	9, 10, 12, 22	Ark Pharm Inc	Ethanol
	MS222	100 mg/L	50 mg/L	10, 12, 29	Sigma - Aldrich	Water
Rainbow Trout	Benzocaine	100 mg/L	50 mg/L	9, 10, 12, 22, 24	Sigma - Aldrich	Ethanol
	Etomidate	2 mg/L	1 mg/L	9, 10, 12, 25	Ark Pharm Inc	Ethanol
	MS222	90 mg/L	45 mg/L	9, 10, 12	Sigma - Aldrich	Water

*Effective dose = Dose at which Stage 5 anaesthesia is achieved. Where the referenced articles cite multiple alternative concentrations, the median was chosen. AstraZeneca do not endorse or recommend any companies listed, other suppliers were available.

No difference was detected in the time spent in the exposure versus control lane during clean water exposure, showing no environmental bias within the system or for the solvent control, suggesting that any effects were driven by avoidance of the anaesthetic compound and not the environment or the solvent carrier. During the study pH within the control and exposure lanes did not differ by more than one unit and it was not considered necessary to buffer all of anaesthetic stocks due to the nature of the compounds and high dilution factor as well as the inherent buffering capacity of the dilution water. PH changes however, do effect the chemistry of the water, and critically the *P*CO_2_ is dependent on pH. Adding anaesthetic compounds to aquarium water may have effects on several physiological parameters such as pH, *p*CO_2_, osmolarity. Changes in these parameters may also induce avoidance or aversive responses. Whilst carbon dioxide is itself often considered an anaesthetic, the pH differences at approximately neutral values used in this study will drive relatively small changes typical of the pragmatic use of the agents and likely not exceed differences experienced in the wild.

A highly significant statistical difference was observed for carp, medaka and rainbow trout when exposed to HCl as a positive control. This combined with a lack of environmental bias in the clean water exposures, suggests that these fish were suitable test species for this behavioural model. The fathead minnow however, showed no response to HCl as a positive control. Indeed 2 (of 20) fish spent 100% of their time in the exposed lane, suggesting either an inability to detect HCl or that the cost benefit associated with moving was considered by the fish to be greater than the effects of the HCl.

The fathead minnow has an established history of use within behavioural testing, use as a species for preference and avoidance testing for aquatic contaminants^14^; avoidance of zinc^15^, and behavioural avoidance of fluoranthene^16^. However, results presented here are difficult to quantify as the positive control using HCl failed to demonstrate an effect. Additionally a statistically significant preference for MS222, was seen with fathead minnow choosing to spend a greater percentage of the experimental time in the exposure lane. In fact six (of 20) fathead minnow chose never to leave the exposure lane. It is not known what elicited this response, or in fact if the response was behavioural or possibly due to greater induction in the anaesthetic than anticipated. We purposefully chose half of the typical induction dose and a relatively short exposure time to minimise potential anaesthesia induction in the test fish, whilst attempting to optimise the relevance to practicable anaesthesia practice. Based on the lack of positive control, the results for fathead minnow are therefore inconclusive and we would suggest the use of an alternative positive control or even test paradigm for use with this species.

Importantly, of the three anaesthetics tested no single anaesthetic elicited the same behavioural responses in all four fish species. Benzocaine elicited a significant avoidance response in medaka, but in none of the other species tested. Etomidate elicited a significant avoidance response in carp, but again in none of the other species tested, MS222 also elicited a significant avoidance response in medaka but in none of the other species tested.

Benzocaine and MS222 have both been used historically as an anaesthetic for all of the species tested suggesting they are an effective anaesthetic. It is proposed that anaesthesia is via a specific mode of action, in the case of benzocaine and MS222 reduced initiation and propagation of action potentials by blocking voltage-sensitive sodium channels^12, 17^. If blocking sodium channels does not induce avoidance in all the species tested, then this behavioural reaction must likely be caused by some other pathway such as some other physical properties of the anaesthetic and not by the induction of anaesthesia. Both benzocaine and MS222 have previously elicited different stress responses when exposed to three different species, Atlantic cod (*Gadus morhua*), Atlantic halibut (*Hippoglossus hippoglossus*) and Atlantic salmon (*Salmo salar*), despite likely having the same mode of action^18^. This would support the contention that avoidance is likely due to some aspect of the physical properties of the compounds; possibly acting as an irritant or aversive to taste/smell receptors.

According to the annual statistics for the use of animals in scientific studies within the UK as released by the Home office (https://www.gov.uk/government/statistics), for the cumulative data available between 2001 and 2013 the UK used a total of 4,622,397 fish in scientific procedures of which 38.3% were exposed to anaesthesia at some part of the study (includes recovery and non-recovery procedures, data on anaesthesia use stopped being collected in 2014). These figures only represent those fish used for scientific procedures, and not fish used in work deemed to be sub threshold i.e. studies not deemed to have the potential to cause pain, suffering, distress or lasting harm, or animals held for husbandry and breeding, or the production of tissues. In practice, we suggest many laboratory fish will experience anaesthesia, but for most fish the first and only exposure to an anaesthetic agent would be during humane killing and euthanasia. Euthanasia is often defined as “a good death” and therefore exposure to any agent used should not elicit avoidance behaviours in order to meet definitions of humane^19^, therefore investigations within this study focussed on initial exposure and not potential behavioural changes caused by repeat exposures. The UK data report that 31.5% of all fish in the 2001 to 2013 period actually underwent recovery, so some may well have gone on to experience anaesthesia for a second time or more, particularly if humanely killed or euthanised using anaesthesia as the first step. This is important if we are to understand the findings that zebrafish can retain an apparent memory of and avoid the place in which they were anaesthetised^5^. For medaka and carp based on the evidence collected here we recommend a humane approach to anaesthesia and first step of euthanasia would be to consider a species specific anaesthetic: avoiding benzocaine in medaka and etomidate for carp. For rainbow trout it appears that all three anaesthetics tested were appropriate.

Within this study three of the species tested were cyprinids (carp, fathead minnow and medaka) and differences were seen between these closely related species, as well as between the fourth species which was a salmonid. In our previous work we found nine anaesthetics induced avoidance for a fourth cyprinid species (zebrafish) and results presented for that species are further supported independently^5^. Reportable differences between three closely related cyprinid species provide evidence for the requirement for species specific anaesthesia to be empirically reviewed by species. The need for individual empirical review is further strengthened by the different stress responses to anaesthetics with the same mode of action within a single fish species^11^.

The fact that MS222 did not elicit avoidance in rainbow trout is contrary to several other publications^20–22^ and also what has been seen anecdotally by the authors. It is possible however, that the lower dose rate used within this study, 50% of the effective dose seen within the literature (Table 2) was not high enough to elicit any behaviours previously seen in these other studies. It may also be the case that higher concentrations of the other agents could also present a relative change in behaviour.

## Conclusions

Within the framework of Directive 2010/63 EU^6^ there is an acceptance that any work undertaken will incorporate the objectives of the 3 R’s (Reduction, Replacement, Refinement). This study presents the evidence for avoidance to commonly used anaesthetics based on one behavioural model, and a limited number of species and strains thereof. Further work is needed within this field to understand behavioural response to anaesthetics within a greater number of species and to identify any strain differences within these species. The use of other behavioural models is also key, in order to fully account for behaviours not easily detected by one system and also to understand the potential lack of suitability of different test species to one particular behavioural paradigm. It is therefore paramount that work undertaken with fish species that involves any element of anaesthesia must be reviewed to ensure that best practice is used commensurable with the aims of the study and that this is done on a species by species basis. Consideration must be given to the morphology and behaviour of individual species as well as the specific aims of the study and should be adopted as best practice for fish anaesthesia. It may also be the case that higher concentrations of the agents could also present a relative change in behaviour, however regardless of the mechanism that induces avoidance, a humane anaesthetic protocol should not elicit an avoidance or aversion response. We also note that the arbitrary restrictions on anaesthetic agents of some countries could make this difficult to implement and so call for international co-operation in establishing best practice.

From the work presented here and in previous submissions^4^; Medaka and zebrafish appear not to avoid etomidate, but avoid benzocaine and MS222. Carp appear not to avoid benzocaine or MS222, but do avoid etomidate. Rainbow trout show no difference in their response to etomidate, benzocaine or MS222. We are unable to draw any conclusions for fathead minnows under this test paradigm.

## Methods

### Ethics Statement

All the experimental *in vivo* protocols and procedures involving fish were performed in accordance with the United Kingdom’s Animals (Scientific Procedures) Act. The *in vivo* studies were undertaken at Brixham Environmental Laboratory, AstraZeneca (United Kingdom) under Project and Personnel Licences granted by the Home Office. All *in vivo* experimental protocols were also approved by the local Ethical Committee at Brixham Environmental Lab in accordance with AstraZeneca’s local and global ethical policies. This work is reported in compliance with the ARRIVE guidelines and details are collated in the ARRIVE supplementary information S1.

### Test species and procedure

Differences in behavioural response to three commonly used anaesthetics were investigated in the following fish species: carp, fathead minnows, medaka and rainbow trout. Twenty animals where used per treatment group (control, positive control, solvent control, benzocaine, MS222 and etomidate) and the study used 480 fish in total (4 species × 20 fish × 6 treatments). Replicate numbers were optimised for the study design from our previous experience with zebrafish^4^.

Application of a chemotaxic chamber to study behavioural aversion to anaesthetics has previously been described in detail^4^, and is outlined briefly here. Individual fish of each species were transferred from stock tanks into the flow by means of a beaker containing a small volume of water (avoiding air exposure). After the transfer, fish were allowed to acclimate for 150 seconds and subsequently a continuous dose of the test compound at a predetermined concentration was introduced via an open port into the top of one of the water inlets that provided the incoming flow for a period of 150 seconds. The inlet was submerged to allow the greatest mixing with the minimum surface disturbance in order to preserve the laminar flow. The side receiving the test compound was alternated for each experimental run using a pre-determined order. The injection of test compound at this point allowed for mixing into the incoming flow, with additional time for mixing in the chamber prior to the baffle. The pH was measured in the mixing chamber in the main body of water to ensure an accurate reading and the final reading was recorded for each exposure in both the clean and exposure lanes. The horizontal gradient created by the laminar flow within the tank allowed the untreated lane to remain uncontaminated, so creating two lanes between which the fish could move freely. Following each experiment with one fish, the system was manually flushed with clean water to remove any test compound residues. The location and activity of the fish with access to both the treated and untreated lane, were recorded via video camera for the whole experimental period. The video camera was positioned directly above the tank, ensuring that the camera did not create any shadowing on the water that could influence lane choice by the test fish. Offline analysis of the video recordings was carried out using VideoTrack analysis software (Version 2.5.0.25, ViewPoint, Lyon, France), and analysed over the 150 second exposure period; the results for each test substance were analysed separately. The data output from VideoTrack was subsequently formatted in Excel (Microsoft office, 2007) for statistical analysis using MLwiN^23^ (available at http://www.bristol.ac.uk/cmm/software/mlwin/). The data were tested against a pre-specified, multilevel model. A multilevel approach was used as it allowed the data structure of the repeated measurements made on each fish (i.e. a measurement for each lane) to be taken into account. A general linear model within the multilevel model then included a term for the effect of treatment lane compared with control lane and also a term for the right hand side of the equipment compared with the left hand side, to ensure that no intrinsic bias was present within the experimental setup. These terms in the model were then tested, using a Chi square statistic, against a change in log likelihood. From the control data, there was no evidence of a left/right flow chamber bias so only the parameter estimates of the effects of the anaesthetic treatments are presented here (Table 1).

### Test substance

Details of the anaesthetics examined in the experiment are listed in Table 2. Each was used at a concentration of 50% of the effective (Table 2), published dose required to produce anaesthesia. All anaesthetic stocks were prepared in accordance with standard practice^12^. To achieve the high stock concentration required for dosing, benzocaine was first solubilised in ethanol. It was not necessary to buffer any of the stocks due to the inherent pH values, this included MS 222. Although MS222 is capable of reducing the pH of dilution water, in this study due to the low dose rate and the inherent buffering capacity of the (OECD) dilution water the pH within the exposure lane never exceeded a difference of more than ±1.0 of that of the control. Effects of a reduction by this amount are deemed to have minimal effects on most fish species^9^.

## Electronic supplementary material

Supplementary Information 
