# Supplementary material for: Species specific anaesthetics for fish anaesthesia and euthanasia
Source: Sci Rep. 2017 Aug 2;7:7102. doi: 10.1038/s41598-017-06917-2 (PMC5541135; doi:10.1038/s41598-017-06917-2)
Supplement: Supplementary file 1 — Supplementary Information [file 41598_2017_6917_MOESM1_ESM.pdf]

Species specific anaesthetics for fish anaesthesia and euthanasia.

Gareth D. Readman a*, Stewart F. Owen b, Toby G. Knowles c, Joanna C. Murrellc

1 School of Biological and Marine sciences, University of Plymouth, Plymouth, United Kingdom, PL4 8AA
2 AstraZeneca, Alderley Park, Macclesfield, United Kingdom , Cheshire, SK10 4TF
3 School of Veterinary Science, University of Bristol, Langford House, Langford, Bristol, United Kingdom, BS40 5DU

***Corresponding author. Present address Plymouth University. Tel.: +44 1752 584474**
Email address: gareth.readman@plymouth.ac.uk (G.D. Readman).

Supplemental information in support of ARRIVE for Readman et al., "Species specific anaesthetics for fish anaesthesia and euthanasia."

Responses to the ARRIVE category checklist available at
https://www.nc3rs.org.uk/sites/default/files/documents/Guidelines/NC3Rs%20ARRIVE%20Guidelines%20Checklist%20%28fillable%29.pdf
Kilkenny C, Browne WJ, Cuthill IC, Emerson M, Altman DG (2010) Improving Bioscience Research Reporting: The ARRIVE Guidelines for Reporting Animal Research. PLoS Biol 8(6): e1000412. doi:10.1371/journal.pbio.1000412

Title 1
Provide as accurate and concise a description of the content of the article as possible.
Species specific anaesthetics for fish anaesthesia and euthanasia.

Abstract 2
Provide an accurate summary of the background, research objectives, including details of the species or strain of animal used, key methods, principal findings and conclusions of the study.
Please refer to the main manuscript.

INTRODUCTION
Background 3
a. Include sufficient scientific background (including relevant references to previous work) to understand the motivation and context for the study, and explain the experimental approach and rationale.
Please refer to the main manuscript.

b. Explain how and why the animal species and model being used can address the scientific objectives and, where appropriate, the study's relevance to human biology.
We wish to characterise the response of four commonly used laboratory fish species to three different anaesthetic agents. There is little relevance to human biology, except that mammalian anaesthesia protocols tend to be species specific, and we hypothesise that

there are likely good reasons why we should apply a similar approach to the much more diverse range of fish used in the laboratory.

Objectives 4
Clearly describe the primary and any secondary objectives of the study, or specific hypotheses being tested.
We wish to characterise the response of four commonly used laboratory fish species to three different anaesthetic agents. Our premise is that fish are able to detect anaesthetic agents in the water and may find them aversive. Our hypothesis is that fish will spend less time in water containing anaesthetic agents than in clean water.

METHODS
Ethical statement 5
Indicate the nature of the ethical review permissions, relevant licences (e.g. Animal [Scientific Procedures] Act 1986), and national or institutional guidelines for the care and use of animals, that cover the research.

All the experimental *in* vivo protocols and procedures involving fish were performed in accordance with the United Kingdom's Animals (Scientific Procedures) Act. The *in vivo* studies were undertaken at Brixham Environmental Laboratory, AstraZeneca (United Kingdom) under Project and Personnel Licences granted by the Home Office. All *in vivo* experimental protocols were also approved by the local Ethical Committee at Brixham Environmental Lab in accordance with AstraZeneca's local and global ethical policies.

Study design 6
For each experiment, give brief details of the study design including:
a. The number of experimental and control groups.
Each experimental and control group consisted of 20 individuals from each of four species exposed to only one of three anaesthetics or three controls (six treatments: control, positive control, solvent control, benzocaine, MS222 and etomidate). Therefore twenty individual fish of four species exposed to three different control plus three compounds (20 x 4 x 6) = 480 fish.

b. Any steps taken to minimise the effects of subjective bias when allocating animals to treatment (e.g. randomisation procedure) and when assessing results (e.g. if done, describe who was blinded and when).
Each fish was selected from a stock of that species where individual differences in size variation had been minimised. Fish were moved into the test arena without air exposure in a water via a beaker and minimal chasing by an experienced operator. The treatment allocation order was pre-determined before the study using a Greco-latin square design to allocate the treatment to the lane of the chemotaxic chamber and therefore a pseudo-randomisation was achieved as the operator could only select the fish before knowing the treatment to be applied. The video tracking removed operator bias in recording the behaviours.

c. The experimental unit (e.g. a single animal, group or cage of animals).

The experimental unit is the individual fish as each was exposed independently to pre-allocated treatment.

A time-line diagram or flow chart can be useful to illustrate how complex study designs were carried out.
The timeline for each species represents a repeated protocol as follows: Allocation of treatment order sequence and lane to which chemical is to be applied; selection of individual fish from stock and transfer to exposure arena; application of treatment to pre-determined lane; acclimation of fish for 150 seconds; recording of behaviour; removal of fish; flushing/cleaning of exposure arena; repeat.

Experimental procedures 7
For each experiment and each experimental group, including controls, provide precise details of all procedures carried out. For example:
a. How (e.g. drug formulation and dose, site and route of administration, anaesthesia and analgesia used [including monitoring], surgical procedure, method of euthanasia). Provide details of any specialist equipment used, including supplier(s).
The chemotaxic chamber was a bespoke construction and detailed in our previous publication Readman, G.D., Owen, S.F., Murrell, J.C., Knowles, T.G. Do fish perceive anaesthetics as aversive? PLoS One. 8, 73773; 10.1371/journal. pone.0073773 (2013).
The solvent and anaesthetics are described in Table 2 of this manuscript.
The video images were analysed using VideoTrack analysis software (Version 2.5.0.25, ViewPoint, Lyon, France), and the data formatted in Excel (Microsoft office, 2007) for statistical analysis using MLwiN23 (available at http://www.bristol.ac.uk/cmm/software/mlwin)

b. When (e.g. time of day).
Exposures were conducted during the day between 5 and 10 hours after artificial sunrise. The fish were all kept under a long day photoperiod of 14h light and the dawn and dusk phasing was 30 minutes long.

c. Where (e.g. home cage, laboratory, water maze).
Fish were maintained in their home husbandry tanks until transfer to the chemotaxic chamber. Fish were not returned to the home stock and were killed following the experimental run.

d. Why (e.g. rationale for choice of specific anaesthetic, route of administration, drug dose used).
This study aimed to test the aversion to three common anaesthetics of four different species of fish. The exposure concentration of each was selected as 50% of a standard dose and detailed in Table 2 of the main manuscript. This exposure was selected to reduce the chance of sedation during the short exposure but allow the fish to respond to a reasonable concentration. Administration was via the water presented in a lamina flow to provide both clean or chemical options to each individual.

Experimental animals

8 a. Provide details of the animals used, including species, strain, sex, developmental stage (e.g. mean or median age plus age range) and weight (e.g. mean or median weight plus weight range).
All fish were selected from stock at the AstraZeneca Brixham Environmental Laboratory, Carp and Rainbow Trout were obtained from commercial UK farms, Fathead minnows were bred in house from stocks obtained from a supply house in the United States and Medaka (HdrR-II1) were bred in house from stocks obtained from a UK university. The names of suppliers are available on request to legitimate scientific investigators. All fish used were of mixed sex and juvenile development. On arrival at the laboratory, all fish undergo a minimum 14d quarantine containment before transfer to the main facility. These stocks were observed as healthy and had been maintained in the main facility.

- Carp (*Cyprinus carpio*), farm supplied, approximately 10 months from hatch. Individual weights were not recorded but the cohort ranged approximately 5 to 6g.
- Fathead minnows (*Pimephales promelas*), 8 months from hatch in house supply. Individual weights were not recorded but the cohort ranged approximately 3 to 4g.
- Medaka (*Oryzias latipes*) 8 months from hatch in house supply. Individual weights were not recorded but the cohort was approximately 1g.
- Rainbow trout (*Oncorhynchus mykiss*) farm supplied, approximately 10 months from hatch. Individual weights were not recorded but the cohort ranged approximately 5 to 6g.

b. Provide further relevant information such as the source of animals, international strain nomenclature, genetic modification status (e.g.knock-out or transgenic), genotype, health/immune status, drug or test naïve, previous procedures, etc.
The source is detailed in 8a above. These fish were, not genetically modified and of unknown genotype. They were test, drug and anaesthetic naïve and had undergone no previous procedures.

Housing and husbandry 9
Provide details of:
a. Housing (type of facility e.g. specific pathogen free [SPF]; type of cage or housing; bedding material; number of cage companions; tank shape and material etc. for fish).
All stocks were classified as conventional stock. These fish were deemed healthy by the trained and experienced operators and showed no symptoms of disease either behaviourally or physically. No additional materials or substrate was provided in their tanks, but enrichment in terms of con-specifics, water movement, as well as this all fish received an optimum diet which in some cases included live foods raised in house to prevent disease transfer and frozen foods that had been irradiated to ensure no comtamination. The range of diets was used both for nutritional reasons and also to increase the range of prey and facilitate a range of behavioural activities.

- Carp (*Cyprinus carpio*), maintained in groups of initially 200 individuals within circular, grey, glass-reinforced plastic (GRP) commercial tanks with 360L volume and 1m diameter. Water was single pass delivered via a spray bar to achieve circulation of the water within the tank to aid orientation of the fish and also aeration, with a flow rate of 3L/min.

- Fathead minnows (*Pimephales promelas*), maintained in single sex groups of 50 adults within a standard glass aquaria 45L volume. Water was single pass with a flow rate of 500ml/min.
- Medaka (*Oryzias latipes*) maintained in mixed sex groups of 50 adults within a standard glass aquaria 45L volume. Water was single pass with a flow rate of 500ml/min.
- Rainbow trout (*Oncorhynchus mykiss*) maintained in groups of initially 200 individuals within circular, grey, glass-reinforced plastic (GRP) commercial tanks with 360L volume and 1m diameter. Water was single pass delivered via a spray bar to achieve circulation of the water within the tank to aid orientation of the fish and also aeration, with a flow rate of 3L/min.

b. Husbandry conditions (e.g. breeding programme, light/dark cycle, temperature, quality of water etc for fish, type of food, access to food and water, environmental enrichment).

The dilution water consisted of potable water dechlorinated by the addition of sodium thiosulphate with secondary removal of any residual chlorine by activated carbon. The controlled addition of salts was used to maintain minimum pH and hardness levels in accordance with (International Organisation for Standardization (ISO), Organisation for Economic Co-operation and Development (OECD), United States Environmental Protection Agency (USEPA)) regulatory testing guidelines (Min/Max figures for daily dilution water recordings during experimental period pH=7.06-7.85 Conductivity 177.7-224µS). The treated water was passed through a set of 20 and 10 µm filters and an ultraviolet steriliser before being delivered to holding tanks prior to distribution to the water system which delivered water at source within the laboratory to the required temperature ± 1ºC. Water delivered to the chemotaxic test chamber was provided from the same supply module for that of the stock to be tested, this therefore ensured no variation in water between the home tank and the testing system. The chemotaxic chamber, as with the entire husbandry unit, used a flow through design, this means that little or no ammonia, nitrite or nitrate is present within the systems. Specifically within the chemotaxic chamber, due to the flow through design and the fact that the fish where exposed for only minutes we expect there to be no ammonia, nitrite or nitrate present. Any ammonia, nitrite or nitrate would be significantly below 0.1mg/L. Osmolarity, alkalinity and hardness were not recorded within the system.

- Carp (*Cyprinus carpio*), farm supplied, 14h light/ 10h dark with 30 minutes of dawn and dusk transition. OECD standard water, 15°C. Fish were fed daily with commercial pellets (BioMar AS, Myre, Norway).
- Fathead minnows (*Pimephales promelas*), 14h light/ 10h dark with 30 minutes of dawn and dusk transition. OECD standard water, 25°C. Fish were fed daily with a combination of commercial pellet (BioMar AS, Myre, Norway) and defrosted irradiated artemia (Gamma Frozen Foods, Tropical Marine Centre, Chorleywood, UK).
- Medaka (*Oryzias latipes*) 14h light/ 10h dark with 30 minutes of dawn and dusk transition. OECD standard water, 28°C. Fish were fed daily with a combination of commercial pellet (ZM Premium granular, ZM Systems, Hampshire, UK), defrosted irradiated artemia (Gamma Frozen Foods, Tropical Marine Centre, Chorleywood,

UK), enriched live artemia (Platinum grade cysts, Argent Chemical Laboratorie Inc. Washington, US, Enriched with SELCO, INVE Aquaculture, Dendermonde, BE).

- Rainbow trout (*Oncorhynchus mykiss*) farm supplied, 14h light/ 10h dark with 30 minutes of dawn and dusk transition. OECD standard water, 15°C. Fish were fed daily with commercial pellets (BioMar AS, Myre, Norway).

Environmental enrichment for stock fish was: provision of water movement via aeration, conspecific group housing; appropriate feeding and live food where species appropriate according to Williams et al 2009 (TD Williams, GD Readman, SF Owen 2009. Key issues concerning environmental enrichment for laboratory-held fish species. Laboratory Animals 43(2), 107-120).

c. Welfare-related assessments and interventions that were carried out prior to, during, or after the experiment.
Fish were routinely observed twice per day and during feeding on three occasions. Observed welfare and health were recorded daily. No welfare interventions were needed and no mortalities recorded in the stocks for at least 2 weeks before any study.

Sample size 10
a. Specify the total number of animals used in each experiment, and the number of animals in each experimental group.
Each experimental and control group consisted of 20 individuals from each of four species exposed to only one of three anaesthetics or three controls (six treatments: control, positive control, solvent control, benzocaine, MS222 and etomidate). No single fish was ever exposed to more than one chemical so this was not a cross-over design. The chemotaxic tank was washed through between fish (see study design 6c above). Therefore twenty individual fish of four species exposed to three different control plus three compounds (20 x 4 x 6) = 480 fish.

b. Explain how the number of animals was arrived at. Provide details of any sample size calculation used.
Our previous publication (Readman et al 2013 cited above) provided an estimate of the variation for zebrafish control data. We anticipated that these new untested species may display different behavioural phenotypes. To accommodate any increase in variability of response with the new species, whilst limiting an increase in the use of animals, sample size was increased by 10. Sample size software does exist for multilevel study designs, however, the number of parameters that were unknown at the start of the study, and whose values would have had to have been assumed, meant that its use would add little information.

c. Indicate the number of independent replications of each experiment, if relevant.
The experiment as described above was run once.

Allocating animals to experimental groups 11
a. Give full details of how animals were allocated to experimental groups, including randomisation or matching if done.
Each fish was selected from a stock of that species where individual differences in size variation had been minimised. Fish were moved into the test arena without air exposure in

a water via a beaker and minimal chasing by an experienced operator. The treatment allocation order was pre-determined using a Greco-Latin square design to randomise which lane of the chamber would be the exposure before the study. Each fish was released into the chemotaxic chamber in the same location in the middle of the tank. The allocation of treatment lane was not concealed as the operator catching the fish knew the treatment to be applied. The video tracking removed operator bias in recording the behaviours.

b. Describe the order in which the animals in the different experimental groups were treated and assessed.
The treatment allocation order was pre-determined before the study.

Experimental outcomes 12
Clearly define the primary and secondary experimental outcomes assessed (e.g. cell death, molecular markers, behavioural changes).
The behavioural response determined by the Viewpoint software was the time spent in the cleanwater lane and the time spent in the treatment lane for each individual.

Statistical methods 13
a. Provide details of the statistical methods used for each analysis.
Analysis was conducted using MLwiN23 (available at http://www.bristol.ac.uk/cmm/software/mlwin) The data were tested against a pre-specified, multilevel model. A multilevel approach was used as it allowed the data structure of the repeated measurements made on each fish (i.e. a measurement for each lane) to be taken into account. A general linear model within the multilevel model then included a term for the effect of treatment lane compared with control lane and also a term for the right hand side of the equipment compared with the left hand side, to ensure that no intrinsic bias was present within the experimental setup. These terms in the model were then tested, using a Chi square statistic, against a change in log likelihood. From the control data, there was no evidence of a left/right flow chamber bias so only the parameter estimates of the effects of the anaesthetic treatments are presented.

b. Specify the unit of analysis for each dataset (e.g. single animal, group of animals, single neuron).
Single animal as each was exposed independently to treatment.

c. Describe any methods used to assess whether the data met the assumptions of the statistical approach.
We carried out graphical assessment of normality of errors and homogeneity of variance, for the analyses and found the distributions showed that the assumptions were met.

RESULTS
Baseline data 14
For each experimental group, report relevant characteristics and health status of animals (e.g. weight, microbiological status, and drug or test naïve) prior to treatment or testing. (This information can often be tabulated).

All fish were observed to be normal and healthy prior to study by trained and experienced aquaculturists. There was no evidence of abnormal colour or behaviour. No mortalities from the parent stock were reported for more than two weeks before study. The animals were test naïve prior to treatment.

Numbers analysed 15
a. Report the number of animals in each group included in each analysis. Report absolute numbers (e.g. 10/20, not 50%).
Twenty fish per treatment group.

b. If any animals or data were not included in the analysis, explain why.
For all animals data was obtained and no animals were excluded from the analysis. The study used and reported 480 individual fish.

Outcomes and estimation 16
Report the results for each analysis carried out, with a measure of precision
(e.g. standard error or confidence interval).
See table 1 of the main manuscript.

Adverse events 17
a. Give details of all important adverse events in each experimental group.
Identification of adverse responses to the anaesthetic in terms of the time spent in or out of the treatment exposure are the primary aim of the manuscript, and are reported extensively through the results and discussion.

b. Describe any modifications to the experimental protocols made to reduce adverse events.
The exposure period was optimised in our previous study with zebrafish and we chose to continue with this short exposure to minimise the length of exposure whilst maintaining the power of the study with an appropriate number of animals. Further, we chose to expose only at half the normal anaesthetic dose typically used in laboratories in order not to induce anaesthesia. In this study paradigm, the fish always have access to clean untreated space. Fish were transferred from stock without netting or air exposure to minimise the handling stress, and both the exposure apparatus and the transfer beaker rinsed between animals to minimise transfer of alarm substances.

DISCUSSION
Interpretation/scientific Implications 18
a. Interpret the results, taking into account the study objectives and hypotheses, current theory and other relevant studies in the literature.
Please refer to the discussion and conclusion of the main manuscript.

b. Comment on the study limitations including any potential sources of bias, any limitations of the animal model, and the imprecision associated with the results.
Please refer to the discussion and conclusion of the main manuscript.

c. Describe any implications of your experimental methods or findings for the replacement, refinement or reduction (the 3Rs) of the use of animals in research.
The aim of this study was to determine the potential adverse response of four species of fish to three commonly used anaesthetic agents and therefore in this case the focus is a 3Rs study. Please refer to the discussion and conclusion of the main manuscript.

Generalisability/ translation 19
Comment on whether, and how, the findings of this study are likely to translate to other species or systems, including any relevance to human biology.
Here we investigated the response of four species of fish to three anaesthetic agents and found significant differences in response among the species. The implication is that care should be taken when choosing anaesthetic agents to work with the many thousands of species of fish as it appears that one anaesthetic agent may not be appropriate for all species.

Funding 20
List all funding sources (including grant number) and the role of the funder(s) in the study.
This work was funded by AstraZeneca under the Safety Health and Environment research programme. AstraZeneca does not have any products marketed or under development as fish anaesthetics. The funder had no role in study design, data collection and analysis, decision to publish, or preparation of the manuscript. The authors have declared that no competing interests exist.
